# The Relationship Between Posttraumatic Stress Disorder and Sleep Quality, Eating Behaviour and Diet Quality in Syrian Migrants: A Cross-Sectional Study

**DOI:** 10.3390/healthcare14070837

**Published:** 2026-03-25

**Authors:** Gülin Öztürk Özkan, Hale Hacıbayram

**Affiliations:** 1 Department of Nutrition and Dietetics, Faculty of Health Sciences, İstanbul Medeniyet University, İstanbul 34862, Turkey; 2 Independent Researcher, Acıbadem Neighbourhood Yaprak Street, No36/4, İstanbul 34660, Turkey; halabaram@gmail.com

**Keywords:** migrants, posttraumatic stress disorder, sleep quality, eating behavior, diet quality

## Abstract

**Background/Objectives:** In recent years, the migrant population has been increasing. Migrants are at risk for malnutrition, mental disorders and related health problems. In this study, we aimed to examine the relationships among diet quality, eating behavior, posttraumatic stress disorders and sleep quality in Syrian migrants. **Methods:** This study included 78 female and 72 male Syrian adult migrants. The participants completed a questionnaire including demographic information, the Pittsburgh Sleep Quality Index, the Posttraumatic Stress Disorder Short Scale, and a three-factor eating questionnaire. For the diet quality calculation, a 24 h retrospective food consumption record was taken. **Results:** A total of 94.0% of the participants had mild to moderate risk of posttraumatic stress disorder. It was determined that 90.7% of Syrian migrants had low or moderate diet quality. There was a positive correlation between the PTSD score and age (r = 0.244) and the Pittsburgh sleep quality index score (r = 0.244) and between the Pittsburgh sleep quality index score and uncontrolled eating (r = 0.171) (*p* < 0.05). The probability of impaired sleep quality increased in individuals with PTSD scores in the T2 (11–19 points) (OR: 1.342; 95% CI: 1.073–1.678) and T3 (20–31 points) (OR: 1.485; 95% CI:1.157–1.905) groups, whereas the probability of improved diet quality increased in individuals in the T2 (11–19 points) (OR: 1.042; 95% CI: 1.000–1.086) group. **Conclusions:** Poor diet quality, risk of posttraumatic stress disorder and poor sleep quality are very common among Syrian migrants. In this respect, evaluating Syrian migrants and taking necessary precautions may help reduce the risk of chronic diseases related to nutrient deficiencies and mental problems. There is a need for policies and programs to manage PTSD among Syrian refugees.

## 1. Introduction

According to the United Nations Refugee Agency, by the end of 2022, 108.4 million people worldwide had to migrate due to persecution, conflict, violence, human rights violations or serious disruption of public order [1]. People may migrate to other countries voluntarily or obligatorily due to dramatic and rapid changes in environmental, economic, political and social structures. Depending on the reasons for migration, people who leave their home countries are given the status of refugee, asylum seeker or migrant. In 2011, when the civil war in Syria started, the number of refugees and asylum seekers migrating to Turkey increased dramatically [2]. Turkey is a transit and final destination for refugees and asylum seekers from many countries worldwide because of its geographical location, cultural structure and welcoming nature [3]. According to official statistics, Turkey hosts the largest number of refugees in the world, with 3.6 million [1]. The most important reasons for this are that Turkey and Syria are neighboring countries and that it is easy to travel to Turkey [2]. At the beginning of the migration wave, asylum-seekers were hosted in Turkey for security reasons, but today, they have become permanent residents [4].

Refugees are exposed to stress not only in their home countries but also in their host countries [5]. Migrants experience extreme stress due to exposure to many traumatic events in their home countries, such as war, witnessing the death of people, generalized violence and torture. As a result of all these events, symptoms of posttraumatic stress disorder (PTSD) can develop. The prevalence of posttraumatic stress disorder among adult refugees has been reported to range from 8 to 63% [6]. In particular, owing to leaving the family, dangerous travel methods, war conditions in the home country, refugee processes, socioeconomic problems, racist attitudes, and posttraumatic stress disorders may persist after migration. This can lead to the development and progression of physical and mental health problems [7]. For these reasons, the prevalence of psychological disorders is greater in migrants than in the general population [8]. A study conducted in Sweden found that 29.9% of Syrian refugees suffer from post-traumatic stress disorder [9].

Sleep has an important role in maintaining physical and mental health. Sleep disorders are prevalent among migrants [10,11]. Sleep disturbance rates among Syrian refugees can reach up to 50% [12]. Various traumatic events and their psychological reactions may contribute to the occurrence of sleep disturbances [11]. Leaving the family and home, the process of adapting to a new and unknown country seems to be a factor that can trigger sleep disorders [13]. Sleep disturbances may contribute to the occurrence or progression of PTSD [14]. The link between PTSD and sleep disturbance in refugee populations is not well known [15]. A study conducted by Sankari et al. [15] found a positive association between PTSD and sleep disturbances among Syrian refugees.

A study conducted by Atayoğlu et al. [16] found that 77.8% of Syrian refugees had poor dietary quality. Migrants are unable to consume adequate food because of difficulties in accessing high-quality and culturally appropriate food. Low income, distance from food outlets and language difficulties are recognized as the most important barriers to food availability. A lack of access to healthy and sufficient food can also negatively affect the quality of the diet. Regardless of the host country, migrants are exposed to hunger and food insecurity. This situation results in many health problems associated with poor diet quality and malnutrition [17]. A healthy diet and diverse food can reduce the risk of developing chronic diseases such as obesity, diabetes, hypertension, myocardial infarction, heart attack and many types of cancer [18].

Posttraumatic stress disorder can be associated with decreased or increased appetite. Uncontrollable stress can change eating patterns and food preferences and trigger the consumption of unhealthy foods rather than healthy foods. In individuals with high stress levels, an increase in the consumption of foods with low nutrients but high energy content can be observed [19]. There are very few studies on stress-induced changes in the eating behavior of refugees. In a study conducted with the participation of Ukrainian refugees, psycho-emotional changes were associated with appetite and unhealthy food intake [19]. Trauma resulting from military interventions can affect eating behavior [20].

There is a relationship between sleep and eating behavior. Food restrictions and increased hunger may negatively affect sleep quality [21]. In addition, poor sleep quality and insufficient sleep also support unhealthy eating behavior [22]. Eating behavior and sleep are regulated by similar neurobiological pathways. This contributes to the strong relationship between eating behavior and sleep [23]. Uncontrolled eating appears to be an important component of the relationship between reduced sleep quality and eating behavior. Uncontrolled eating exacerbates sleep disturbances [22].

Syrian refugees were forced to leave their country due to the conflicts there. The conflict environment and forced migration had significant effects on individuals of all age groups. This study was conducted to identify stress disorders related to the trauma experienced by individuals who were forced to leave their countries due to conflicts and to examine the relationship between post-traumatic stress disorder and sleep disorders, eating behavior, and diet quality. Based on the study results, it is intended to make recommendations for the assessment of refugees in terms of post-traumatic stress disorder to prevent the possible consequences of post-traumatic stress disorder in Syrian refugees.

## 2. Material and Methods

This study was conducted between April and June 2023 with the participation of 150 adult Syrian refugees. Prior to commencing the study, a G*Power (3.1.9.7 for Windows) analysis (efect size 0.3, Power 0.95) was conducted, taking into account similar studies, and it was determined that the sample size should be at least 111. To increase the reliability of the study, 150 adult individuals were included in the study. The aim was to have an equal number of females and males in the sample. However, due to the reluctance and hesitation of women to participate in the study, only 78 women could be included. The study was conducted with the participation of adult individuals who had migrated from Syria following the war, had no diagnosed psychological disorders, had not been diagnosed with an eating disorder, had not been diagnosed with a sleep disorder, and were willing to participate in the study. Non-voluntary Syrian migrants, migrants who had left Syria prior to the war, individuals who had migrated from countries other than Syria, the elderly, children and infants, individuals with a diagnosed psychological disorder, those diagnosed with an eating disorder, those diagnosed with a sleep disorder, individuals whose medical or mental condition made them unsuitable for the survey, and non-migrants were excluded from the study.

In Istanbul, the regions where Syrian migrants live densely were visited, and adults were invited to participate in the study. Health facilities in areas where migrants live were included in the study by visiting social spaces where migrants spend time and randomly selecting adult volunteers. The author, a Syrian refugee, shared information about the study on social media and invited Syrian adult refugees living in Istanbul to participate. Face-to-face questionnaires were administered to refugees who responded positively to the invitation. Written informed consent was obtained from the individuals who agreed to participate in the study. One of the authors is a Syrian migrant, and his first language is Arabic. The questions included in the questionnaires and scales were asked to the participants in Arabic by the author.

A face-to-face questionnaire including 8 questions about demographic and general information and height and weight measurements was administered to the participants. The questionnaire also included the Pittsburgh Sleep Quality Index, posttraumatic stress disorder short scale, and three-factor eating questionnaire. In addition, retrospective 24 h food consumption records of the patients were taken. The healthy eating index was calculated via food consumption records, and diet quality levels were determined. Body mass index (BMI) values were calculated by measuring the height and body weight of the participants. Body mass index was calculated via the formula body weight (kg)/[height (m)]^2^. A body mass index < 18.5 kg/m^2^ is classified as underweight, 18.5–24.9 kg/m^2^ as normal weight, 25.0–29.9 kg/m^2^ as overweight and ≥30 kg/m^2^ as obese [24].

### 2.1. Pittsburgh Sleep Quality Index (PSQI)

This scale is widely used to assess sleep disturbances. The translation of the scale into Arabic was carried out by Suleiman and et al. in 2010, and the Cronbach alpha value for the scale was calculated as 0.65 [25]. The Pittsburgh Sleep Quality Index (PSQI) is a scale used to assess sleep quality over the past month. The scale includes 19 items (for example, during the past month, how would you rate your sleep quality overall?) and seven components: subjective sleep quality, sleep latency, sleep duration, habitual sleep efficiency, sleep disturbance, sleep medication use and daytime dysfunction. The score obtained from all these components is the global scale score and ranges between 0 and 21 points. If the global score is <5, it is determined that sleep quality is good, and if it is ≥5, it is determined that sleep quality is poor [26].

### 2.2. Posttraumatic Stress Disorder Short Scale (PTSD-SS)

The National Stressful Events Survey for PTSD-Short Scale was originally created by LeBeau et al. in 2014 to describe posttraumatic stress disorder [27]. The Posttraumatic Stress Disorder Short Scale was used to assess the participants’ posttraumatic stress disorder status. The scale consists of 9 questions (for example, when something reminds you of a stressful experience, you feel emotionally quite bad). A 5-point Likert scoring system was used to score the answers. The answers are scored from not at all (0 points) to all the time (4 points). The total score varies between 0 and 36. The cutoff score for the PTSD-SS is 24 points. Individuals scoring above 24 points are considered to have a high risk of posttraumatic stress disorder [27,28]. An increase in the total score indicates an increase in the severity of posttraumatic stress. Classification is performed according to the average score obtained by dividing the total score by the number of items. A mean score of 0 means none, 1 means mild, 2 means moderate, 3 means severe and 4 means extreme [29].

### 2.3. Three-Factor Eating Questionnaire (TFEQ-R18)

The Three-Factor Eating Questionnaire (TFEQ) consists of 3 subfactors: uncontrolled eating (for example, sometimes when I start eating, I just can’t seem to stop), cognitive restraint (for example, I don’t eat some foods because they make me fat) and emotional eating (for example, I start to eat when I feel anxious). The uncontrolled eating score ranged from 9 to 36, the cognitive restraint score ranged from 6 to 24, and the emotional eating score ranged from 6 to 24. The higher the score obtained from each subgroup of the scale is, the more likely it is that the individual is under the influence of eating behavior related to that factor [30]. The Arabic validity and reliability study of this scale was conducted by Alhesbi et al. Cronbach’s alpha values were calculated as 0.778 for the uncontrolled eating subscale, 0.588 for the cognitive restraint subscale, and 0.784 for the emotional eating subscale [31].

### 2.4. Healthy Eating Index-2015

The healthy eating index is used to assess diet quality [32]. To calculate the healthy eating index score, 24 h food consumption records were taken retrospectively, and the daily consumption amounts of the healthy eating index components were calculated. All foods in the retrospective 24 h food consumption record were recorded in the BeBIS program, and daily amounts were obtained. The healthy eating index-2015 (HEI-2015) was calculated on the basis of daily food quantities, and diet quality was determined. HEI-2015 consists of a total of 13 dietary components (whole grains, dairy products, total fruit, whole fruit, total vegetables, total protein foods, greens and beans, seafood and vegetable proteins, fatty acids, added sugars, refined grains, saturated fats and sodium). The total score for the healthy eating index-2015 ranges from 0 to 100. A score of 0–50 indicates poor diet quality, a score of 51–80 indicates improved diet quality, and a score > 80 indicates good diet quality [33].

The IBM SPSS Statistics22 (Statistical Package for the Social Sciences) package program was used to evaluate the data obtained in the study. Descriptive statistical methods (means, standard deviations, medians, frequencies, percentages) were used to evaluate the study data. In addition, a chi-square test was also performed to examine the differences according to sex for qualitative data. Independent samples t tests and one-way ANOVA were used to evaluate the differences between the means of the quantitative data. The conformity of the quantitative data to a normal distribution was tested via the Shapiro-Wilk test and graphical analysis. Pearson correlation and multinomial regression analyses were used to evaluate the relationships between quantitative variables. Statistical significance was accepted at *p* ≤ 0.05.

## 3. Results

### 3.1. Participants’ General Characteristics

This study was conducted between April 2023 and June 2023 and included 150 Syrian migrants, who were 48.0% (n = 72) males and 52.0% (n = 78) females. The mean age of the participants was 29.5 ± 10.5 years, and the mean body mass index (BMI) was 24.7 ± 5.1 kg/m^2^ (Table 1).

Table 1 shows the demographic characteristics of the Syrian migrants and their assessments of diet quality, eating behaviors and posttraumatic stress disorders. A total of 59.7% of the males (n = 43) were employed, whereas 71.8% of the females (n = 56) were not employed. A total of 84.7% of the males (n = 61) and 56.4% of the females (n = 44) were single. It was determined that 88.9% of the males (n = 64) and 61.5% of the females (n = 48) did not have children (Table 1). This finding indicates that the Syrian refugees participating in the study are largely composed of individuals who are independent and have no dependents.

### 3.2. Diet Quality and Eating Behavior

A total of 93.1% of the males (n = 67) and 88.5% of the females (n = 69) had poor diet quality. This study found that nearly all participants had poor dietary quality. This finding is particularly striking and indicates that nearly all Syrian adult refugees are at risk in terms of nutrition. A total of 37.5% of the males (n = 27) and 48.7% of the females (n = 38) were under the influence of cognitive restriction behavior (Table 1). When individuals’ eating behaviors were also assessed, it was found that cognitive restrictive behaviors were prevalent, with women being at greater risk.

### 3.3. Participants’ Posttraumatic Stress Disorder

Sixty percent (n = 90) of the participants had a mild risk of posttraumatic stress disorder, and 34.0% (n = 51) had a moderate risk of posttraumatic stress disorder. It was determined that 73.6% of males (n = 53) and 47.4% of females had a mild risk of posttraumatic stress disorder (Table 1). Nearly all participants exhibited posttraumatic stress disorder. The prevalence of this disorder indicates that it is a significant problem requiring assessment, that its causes must be investigated, and that policies for its management need to be developed.

### 3.4. Posttraumatic Stress Disorder and General Characteristics

Table 2 shows the distributions of the general characteristics and diet quality levels of Syrian migrants according to posttraumatic stress disorder score tertiles. A total of 44.4% (n = 32) of the males and 40.0% (n = 42) of the singles were in the T1 group, 41.0% (n = 32) of the females, 46.7% (n = 21) of the married individuals and 50.0% (n = 19) of the individuals who had children were in the T3 group (*p* < 0.05). These findings suggest that family and gender may be determining factors for PTSD. The high proportion of married individuals with children in the group with the highest level of posttraumatic stress (T3) indicates that an increase in responsibilities may elevate stress levels. This suggests that migrants who are married and have children should be included in mental health improvement initiatives.

### 3.5. Posttraumatic Stress Disorder, Age and Sleep Quality

Table 3 shows the mean age, body mass indices, eating behavior scores, Pittsburgh sleep quality index scores and healthy eating index-2015 scores of the participants according to posttraumatic stress disorder score tertiles. The mean age (32.9 ± 10.6) and mean Pittsburgh sleep quality index score (14.3 ± 2.0) of the individuals in the T3 group were significantly greater than those of the individuals in the T1 group (26.8 ± 9.4) (13.3 ± 1.8) (*p* < 0.05). The fact that the group with the highest average age also had the highest rates of PTSD may be due to their longer exposure to conflict and/or having experienced greater loss. It was observed that participants’ sleep quality deteriorated in parallel with their PTSD levels. It can be concluded that age and sleep disturbances are significant predictors of PTSD among Syrian refugees. This finding suggests that age and sleep disturbances should also be considered in the management of PTSD.

### 3.6. Relationship Between Posttraumatic Stress Disorder, Age and Sleep Quality

Table 4 shows the results of the correlation analysis for the relationships between posttraumatic stress disorder and the Pittsburgh sleep quality index scores, age, body mass indices, eating behavior scores and healthy eating index-2015 scores of Syrian immigrants. The PTSD score was positively correlated with age (r = 0.244) and the Pittsburgh Sleep Quality Index score (r = 0.244) (*p* < 0.05). This finding demonstrates the association between advancing age and deteriorating sleep quality with increased severity of posttraumatic stress disorder symptoms.

### 3.7. Relationship Between Sleep Quality and Eating Behavior

A positive correlation was also observed between the Pittsburgh sleep quality index score and uncontrolled eating (r = 0.171) (*p* < 0.05) (Table 4). This finding indicates that impaired sleep quality negatively affects control over food intake.

### 3.8. Posttraumatic Stress Disorder, Sleep Quality and Diet Quality

Table 5 shows the results of the multinomial regression analysis for the participants’ general characteristics, eating behavior scores, body mass indices, healthy eating index-2015 scores and posttraumatic stress disorder score tertiles. Individuals with posttraumatic stress disorder scores in the T2 (OR: 1.342; 95% CI: 1.073–1.678) and T3 (OR: 1.485; 95% CI: 1.157–1.905) groups were more likely to have increased PSQI scores. The regression model indicates that an increase in post-traumatic stress disorder (PTSD) is significantly associated with a higher likelihood of poor sleep quality (*p* < 0.05). Compared to individuals in the group with mild stress level (T1), those with moderate (T2) and severe (T3) stress levels showed increases in PSQI scores of 1.34 and 1.48 times, respectively. The likelihood of an increase in healthy eating index-2015 scores increased in individuals with a posttraumatic stress disorder score in the T2 group (OR: 1.042; 95% CI: 1.000–1.086) (*p* < 0.05). This result indicates that dietary quality is better in individuals with moderate (T2) PTSD compared to those with mild (T1) PTSD. The fact that the confidence intervals are close to 1 suggests that the relationship is weak and implies the presence of other confounding factors not captured in this analysis.

## 4. Discussion

There are people all over the world who have been forced to leave their homeland and migrate for various reasons. Turkey is a country that hosts immigrants from many different countries [16]. This study was conducted to evaluate the diet quality of Syrian refugees and to examine the relationships between PTSD and sleep quality.

### 4.1. General Characteristics of Participants

Since the study focuses on a disadvantaged group and general characteristics can affect variables, the general characteristics of the sample were also examined. A study involving Syrian refugees living in refugee camps revealed that 40.0% of women and 33.0% of men were primary school graduates, whereas 2.4% of women and 13.2% of men were university graduates [34]. In this study, 76.9% of the females and 63.9% of the males were university graduates (Table 1). The educational level of the Syrian refugees and asylum seekers who participated in this study was found to be higher than that of the Syrian refugees in the previous study [34]. Although both studies were conducted in Turkey, this difference was thought to be due to the difference between staying in a refugee camp and living in the city. Despite the high level of education, women’s participation in the workforce is low. This situation brings to mind the phenomenon of brain drain and points to employment problems in the host country. The failure to participate in the workforce despite a high level of education may contribute to a negative impact on psychological well-being.

In a study conducted by Sağaltıcı et al. [34] with the participation of Syrian refugees, 11.2% of females and 1.6% of males were not employed. In this study, 71.8% of the females (n = 56) and 40.3% of the males (n = 29) were not working (Table 1). In this study, as in the previous study, the employment rate of females was found to be lower than that of males. In this respect, both studies support each other. In this study, 11.1% (n = 8) of the males and 38.5% (n = 30) of the females had children (Table 1) (*p* < 0.05). One of the reasons why women are less involved in occupational life may be the obligation to take care of their children. These studies show once again that the participation rate of females in working life is low, especially in underdeveloped and developing countries. Low employment rates among women may hinder their integration into the host country and their economic independence, potentially leading to an increased likelihood of PTSD development or an exacerbation of existing PTSD symptoms.

A previous study revealed that 2.4% of the migrants were underweight, 45.2% were normal weight, 33.7% were slightly overweight, and 18.7% were obese [35]. In this study, 6.7% of the Syrian migrants (n = 10) were underweight, 52.7% (n = 79) were normal weight, 28.0% were overweight (n = 42), and 12.7% were obese (n = 19) (Table 1). In both studies, the body weights of migrants were concentrated in the normal and overweight categories. It is expected that the proportion of underweight individuals among migrants is greater. However, these results show that malnutrition among migrants is not very common, contrary to popular belief.

### 4.2. Diet Quality

It was determined that refugees were malnourished in terms of most micronutrients [36]. Poor diet quality can lead to nutrient deficiencies [37]. In another study, the diet quality of 77.75% of Syrian refugees was determined to be low, and 22.52% of those refugees were at a level that needed improvement [16]. In this study, 90.7% of Syrian migrants (n = 136) had poor diet quality, 9.3% (n = 14) had diet quality at a level that needed improvement, and no participants had good diet quality (Table 1). The widespread prevalence of poor dietary quality may stem from low labor force participation rates, the high cost of urban living, and the inability to access sufficient quantities of healthy food due to economic constraints. This situation may be linked to the fact that malnutrition among migrants is associated with being forced to leave their home countries, jobs, and lives.

### 4.3. Eating Behavior

In this study, 43.3% of Syrian migrants (n = 65) were under the influence of cognitive restriction behavior. A total of 48.7% of the females (n = 38) and 37.5% of the males (n = 27) were under the influence of cognitive restriction behavior (Table 1) (*p* < 0.05). The BMI values of the participants may have affected their eating behavior. In this study, 37.5% (n = 27) of the males and 43.6% (n = 34) of the females were overweight or obese (Table 1). BMI values may also have an effect on the increased prevalence of CRS behavior. There are studies evaluating immigrants’ eating habits, food cooking methods and diet quality [16,38]. However, there is a lack of research on the dietary behaviors of Syrian refugees, creating a significant gap in refugee health research. Assessing psychological stress is crucial for increasing the effectiveness of dietary interventions.

### 4.4. Posttraumatic Stress Disorder

The rate of PTSD is higher among refugees than among the general population [39]. Millions of refugees worldwide suffer from posttraumatic stress disorder [40]. In studies conducted in different countries, between 29.7% and 61.0% of immigrants and asylum seekers have been observed to have PTSD [8,41]. In a study conducted by Minihan et al. [5] in Australia, 36.6% of the refugees had a high risk of PTSD, and 23.0% had a moderate risk of PTSD. In this study, 60.0% of Syrian migrants had a mild risk of PTSD, and 34.0% had a severe risk of PTSD (Table 1). All of these research findings indicate that PTSD is prevalent among migrants and that its prevalence may vary depending on the destination country. Factors that may arise after migration, such as housing problems, economic circumstances, and legal uncertainties, can contribute to chronic stress. Post-traumatic stress disorder may also increase the risk of developing various psychological and physiological problems.

In a study conducted by Kéri [42] with the participation of refugees, 57.0% of males and 72.0% of females were diagnosed with posttraumatic stress disorder. In another study, 53.0% of female refugees and 47.0% of male refugees were found to have PTSD [43]. In a study conducted by Solberg et al. [8], 48.2% of female refugees and 65.4% of male refugees had posttraumatic stress disorder. In the present study, 44.4% (n = 32) of the males were in the T1 group, and 41.0% (n = 32) of the females were in the T3 group (Table 2) (*p* < 0.05). Although contradictory results have been reported, posttraumatic stress disorder is more common and more severe in females. This may be because females are more sensitive to society than males are.

Marital status may also affect the presence of PTSD. A total of 16.7% of single Syrian migrants and 50.5% of married migrants had PTSD [44]. In this study, 40.0% (n = 42) of single migrants had PTSD scores in the T1 group, and 46.7% (n = 21) of married migrants had PTSD scores in the T3 group (Table 2) (*p* < 0.05). These results suggest that marital status may be related to the presence and degree of posttraumatic stress disorder. Posttraumatic stress disorder may occur more frequently and more severely in married individuals.

Refugees who are parents have been observed to have more PTSD symptoms than their children [45]. Another study reported that immigrants who have children experience post migration stress from their children [46]. In this study, 50.0% of the individuals who had children and 23.2% of those who did not have children had PTSD scores in the T3 group (Table 2) (*p* < 0.05). These findings indicate that family responsibilities and the effort to ensure children’s safety in a foreign country can contribute to the development of stress. Efforts to protect children from the negative effects of migration can place pressure on parents. This study underscores the necessity of establishing centers for the protection of migrants’ mental health that include provisions for all family members.

Research results investigating the relationship between age and posttraumatic stress dysregulation are contradictory [47]. The rate of PTSD increases with age in females, whereas the rate of PTSD decreases with age in males [48]. In their study, Alpak et al. [49] reported that the rate of PTSD in Syrian refugees aged 40 years and younger (36.5%) was higher than that in individuals over 40 years of age (28.6%). In this study, the mean age of individuals with PTSD scores in the T3 group (32.9 ± 10.6 years) was greater than that in the T1 group (26.8 ± 9.4 years) (Table 3) (*p* < 0.05). In the present study, a positive correlation was found between age and PTSD score (Table 4) (r = 0.244). As a result, age can contribute to PTSD, and the level of PTSD increases with increasing age.

### 4.5. Sleep Quality

Sleep disturbance is very common among migrants, ranging from 23.0% to 75.5% [50,51]. In one study, the percentage of Syrian and Iraqi refugees with possible insomnia was 32.6%, and the percentage of individuals with insomnia was 32.6% [52]. In another study, 33.63% of Syrian refugees were found to have sleep disorders [15]. In this study, 99.3% of Syrian migrants had poor sleep quality (Table 1). Previous studies have shown that sleep disorders are common among immigrants. In this study, migrants were determined to have poor sleep quality. In this respect, it supports other studies. However, in this study, almost all the participants experienced poor sleep quality.

### 4.6. Relationship Between Posttraumatic Stress Disorder and Sleep Quality

Migrants are at risk of sleep problems. The mean PSQI score of Syrian refugees was 5.15 ± 2.49 [15]. The mean PSQI score of Syrian refugee women was 6.79 ± 3.57, whereas that of men was 5.93 ± 3.4 [11]. In this study, the mean PSQI score was 13.9 ± 2.1 for men and 13.8 ± 1.9 for women (Table 1). This result indicates worse sleep quality than the results of other studies. Refugees may have health problems in the future due to poor sleep quality.

Sleep disturbance is very common among migrants and refugees and is associated with PTSD [13,53]. In the present study, the individuals with the highest mean PSQI score were in the T3 group (14.3 ± 2.0), and the difference in the mean PSQI score between the T1 and T3 groups was significant (Table 3) (*p* < 0.05). Sleep quality decreases with increasing PSQI score [54]. Individuals with the poorest sleep quality were individuals with severe posttraumatic stress disorder.

Sleep disturbances are reportedly associated with PTSD [13]. Severe mental health symptoms, such as posttraumatic stress disorder, are positively correlated with sleep disturbance [39]. A positive relationship between posttraumatic stress disorder and sleep disturbance has been reported [15]. In this study, a positive correlation was found between posttraumatic stress disorder and the PSQI scores of Syrian refugees (r = 0.244) (Table 4). This result shows that posttraumatic stress disorder negatively affects sleep quality. In this study, the mean PSQI score was more likely to increase in individuals with PTSD scores in the T2 (OR: 1.342; 95% CI: 1.073–1.678) and T3 (OR: 1.485; 95% CI: 1.157–1.905) groups (Table 5) (*p* < 0.05). These results suggest that PTSD may be effective in treating sleep disorders in Syrian migrants. Sleep disorder is a significant health problem and is positively associated with PTSD. Trauma negatively affects sleep quality by impairing REM sleep quality [55]. Improving sleep quality can be considered an important component of trauma recovery. Sleep disorders associated with PTSD in migrants may contribute to health problems that may arise due to sleep disorders in the future and may negatively affect public health.

### 4.7. Relationship Between Eating Behaviors and Sleep Quality

Eating behaviors are associated with sleep quality [56]. In one study, emotional eating behavior was positively associated with sleep quality (r = 0.190) but not with uncontrolled eating (r = 0.110) or cognitive restraint (r = 0.05) [57]. It was determined that there was a significant relationship between uncontrolled eating and emotional eating and sleep quality [22]. In the present study, uncontrolled eating behavior and the PSQI score were positively related (r = 0.171) (Table 4). According to this study, uncontrolled eating behavior may decrease sleep quality. There are conflicting study results explaining the relationship between eating behavior and sleep quality. These results suggest that there may be a relationship between uncontrolled eating and sleep quality disorders. Insufficient and poor-quality sleep may be one of the triggers for uncontrolled eating habits. Identifying and treating sleep disturbances during the nutritional intervention process may positively influence the success of the intervention.

### 4.8. Relationship Between Posttraumatic Stress Disorder and Diet Quality

In this study, Syrian migrants with PTSD scores in the T2 group were more likely to have increased HEI-2015 scores than those in the T1 group (OR: 1.047; 95% CI: 1.000–1.086) (*p* < 0.05) (Table 5). This result shows that an increase in the PTSD level in the T2 group increases the likelihood of an increase in diet quality. However, this increase was not observed in the T3 group (*p* > 0.05). This finding suggests that posttraumatic stress disorder can lead migrants to improve their diet quality to a certain degree. However, migrants with very high PTSD levels are not interested in improving their diet quality. However, no studies have focused on the relationship between posttraumatic stress disorder and diet quality. There is a need for extensive research on this subject.

## 5. Conclusions

Turkey is also one of the largest recipient countries of migrants and hosts the largest number of Syrian refugees in the world. This study revealed that the dietary quality of all Syrian refugees is inadequate and needs to be improved. When dietary behaviors were assessed, it was found that cognitive restrictive behavior was more prevalent than other dietary behaviors. Nearly all migrants have PTSD. Additionally, it was found that all participants had poor sleep quality. Post-traumatic stress disorder is more prevalent and severe among women compared to men, among married individuals compared to singles, and among those with children compared to those without. Migrants with high levels of PTSD were older and had poorer sleep quality compared to those with low levels. There is a positive association between post-traumatic stress disorder, age, and sleep disturbances. Uncontrolled eating behavior is associated with reduced sleep quality. This study provides important findings for the development of public health policies. The prevalence of poor dietary quality and sleep disturbances among Syrian refugees indicates the need to expand health screenings for refugees in these areas. Additionally, given that PTSD is a significant health issue among Syrian refugees, policies must be developed to assess stress levels and manage stress, screening programs must be established, and psychological support units must be created within health centers serving refugees. Since long-term employment and economic difficulties can exacerbate PTSD, the development of social policies addressing these issues and ensuring the labor force participation of educated migrants will lay the groundwork for reducing stress by supporting social integration.

Limitations: The fact that the study was conducted in a single city and involved a limited number of adult migrants limits the generalizability of the findings. To increase the generalizability of the findings, the study should be repeated with a larger sample. The results of this study indicate that posttraumatic stress disorder is prevalent among Syrian refugees and can affect sleep quality. It has also been determined that sleep quality can affect uncontrolled eating behavior. The fact that no validation study has been conducted for the Arabic version of the short scale for posttraumatic stress disorder may constitute a limitation. The finding that posttraumatic stress disorder is prevalent indicates that individuals’ mental states need to be carefully monitored. It is important for Syrian refugees to undergo psychological assessment and for psychological support policies to be established in order to protect public health. This would make it possible to prevent the progression of various disorders that may arise due to trauma-related stress and prevent adverse physiological consequences, ultimately contributing to the protection and improvement of public health.

## Figures and Tables

**Table 1:** Participants’ general characteristics, diet quality, eating behavior and posttraumatic stress disorder status.

	Total	Male	Female	χ^2^	*p*
**Participants (n (%))**	150 (100.0)	72 (48.0)	78 (52.0)		
**Education (n (%))**					
Primary school/middle school	1 (0.7)	0 (0.0)	1 (1.3)	4.537	0.209
High school	36 (24.0)	22 (30.6)	14 (17.9)		
University	106 (70.7)	46 (63.9)	60 (76.9)		
Postgraduate	7 (4.7)	4 (5.6)	3 (3.8)		
**Employment Status (n (%))**					
Yes	65 (43.3)	43 (59.7)	22 (28.2)	15.145	0.000 ^a,^*
No	85 (56.7)	29 (40.3)	56 (71.8)		
**Marital Status (n (%))**					
Single	105 (70.0)	61 (84.7)	44 (56.4)	14.291	0.000 ^a,^*
Married	45 (30.0)	11 (15.3)	34 (43.6)		
**Do you have children? (n (%))**					
Yes	38 (25.3)	8 (11.1)	30 (38.5)	14.806	0.000 ^a,^*
No	112 (74.7)	64 (88.9)	48 (61.5)		
**Body Mass Index (BMI) (n (%))**					
Underweight	10 (6.7)	3 (4.2)	7 (9.0)	2.590	0.459
Normal	79 (52.7)	42 (58.8)	37 (47.4)		
Overweight	42 (28.0)	18 (25.0)	24 (30.8)		
Obesity	19 (12.7)	9 (12.5)	10 (12.8)		
**Diet Quality (n (%))**					
Poor	136 (90.7)	67 (93.1)	69 (88.5)	0.934	0.248
Needs to be improved	14 (9.3)	5 (6.9)	9 (11.5)		
**Eating Behavior (n (%))**					
Uncontrolled eating	34 (22.7)	23 (31.9)	11 (14.1)	6.829	0.033 ^a,^*
Cognitive restraint	65 (43.3)	27 (37.5)	38 (48.7)		
Emotional eating	51 (34.0)	22 (30.6)	29 (37.2)		
**Posttraumatic Stress Disorder (n (%))**					
None	1 (0.7)	1 (1.4)	0 (0.0)	12.800	0.005 ^a,^*
Mild	90 (60.0)	53 (73.6)	37 (47.4)		
Moderate	51 (34.0)	15 (20.8)	36 (46.2)		
Severe	8 (5.3)	3 (4.2)	5 (6.4)		
	**Mean (SD)**	**Mean (SD)**	**Mean (SD)**		*p*
Age (years)	29.5 (10.5)	26.6 (8.3)	32.3 (11.6)		0.001 ^b,^*
Body Mass Index (BMI) (kg/m^2^)	24.7 (5.1)	24.9 (5.6)	24.5 (4.7)		0.575
Pittsburgh Sleep Quality Index score	13.8 (2.1)	13.9 (2.1)	13.8 (1.9)		0.674

^a^ Pearson chi-square test; ^b^ Independent samples *t* test; * *p* < 0.05.

**Table 2:** General characteristics of participants and their diet quality status according to posttraumatic stress disorder tertiles.

	Posttraumatic Stress Disorder Score			
	T1 (0–10 Points)	T2 (11–19 Points)	T3 (20–31 Points)	*p*
**Gender (n (%))**				
Male	32 (44.4)	27 (37.5)	13 (18.1)	0.005 *
Female	20 (25.6)	26 (33.3)	32 (41.0)	
**Education (n (%))**				
Primary school/middle school/high school	15 (40.5)	15 (40.5)	7 (18.9)	0.237
University/Postgraduate	37 (32.7)	38 (33.6)	38 (33.6)	
**Employment Status (n (%))**				
Yes	23 (35.4)	24 (36.9)	18 (27.7)	0.860
No	29 (34.1)	29 (34.1)	27 (31.8)	
**Marital Status (n (%))**				
Single	42 (40.0)	39 (37.1)	24 (22.9)	0.010 *
Married	10 (22.2)	14 (31.1)	21 (46.7)	
**Do you have children? (n (%))**				
Yes	9 (23.7)	10 (26.3)	19 (50.0)	0.008 *
No	43 (38.4)	43 (38.4)	26 (23.2)	
**Body Mass Index (BMI) (n(%))**				
Underweight	3 (30.0)	4 (40.0)	3 (30.0)	0.828
Normal	28 (35.4)	26 (32.9)	25 (31.6)	
Overweight	17 (40.5)	15 (35.7)	10 (23.8)	
Obesity	4 (21.1)	8 (42.1)	7 (36.8)	
**Diet Quality (n (%))**				
Poor	48 (35.3)	48 (35.3)	40 (29.4)	0.846
Needs to be improved	4 (28.6)	5 (35.7)	5 (35.7)	

Pearson chi-square test; * *p* < 0.05.

**Table 3:** Average age, body mass index score and scale score according to posttraumatic stress disorder tertiles.

	Posttraumatic Stress Disorder Score Tertiles				
	T1 (0–10 Points)	T2 (11–19 Points)	T3 (20–31 Points)	*p*	Post Hoc Test
**Age (Years) (Mean(SD))**	26.8 (9.4)	29.4 (10.8)	32.9 (10.6)	0.016 ^a,^*	T3 > T1 (*p* = 0.008)
**Body Mass Index (BMI)** **(kg/m^2^) (Mean (SD))**	25.0 (5.8)	24.5 (4.7)	24.7 (4.9)	0.897	
**Uncontrolled eating score (Mean (SD))**	39.2 (20.5)	34.9 (19.7)	35.8 (17.7)	0.496	
**Cognitive restraint score (Mean (SD))**	46.5 (21.3)	48.0 (21.6)	50.9 (24.9)	0.626	
**Emotional eating score (Mean (SD))**	38.5 (32.9)	35.2 (35.7)	41.5 (38.1)	0.684	
**Pittsburgh Sleep Quality Index Score (Mean (SD))**	13.3 (1.8)	14.0 (2.2)	14.3 (2.0)	0.024 ^a,^*	T3 > T1 (*p* = 0.017)
**Healthy Eating Index Score (Mean (SD))**	33.0 (11.5)	37.0 (9.8)	36.3 (10.8)	0.133	

^a^ One-way ANOVA; Post Hoc Test (Tukey); * *p* < 0.05.

**Table 4:** Correlation analysis.

	Posttraumatic Stress Disorder		Pittsburgh Sleep Quality Index	
	r	*p*	r	*p*
**Age**	0.244 **	0.003	−0.098	0.232
**Body Mass Index (BMI)**	−0.023	0.779	−0.069	0.403
**Uncontrolled eating**	−0.067	0.415	0.171 *	0.036
**Cognitive restraint**	0.143	0.081	−0.136	0.097
**Emotional eating**	0.038	0.643	0.121	0.139
**Pittsburgh Sleep Quality Index**	0.244 **	0.003	1	
**Healthy Eating Index-2015**	0.157	0.056	−0.075	0.362

Pearson correlation test, * *p* < 0.05, ** *p* < 0.01.

**Table 5:** Multinomial regression analysis.

	Posttraumatic Stress Disorder					
	T1 vs. T2			T1 vs. T3		
	OR	%95 CI	*p*	OR	%95 CI	*p*
**Age**	1.056	0.984–1.133	0.128	1.053	0.980–1.131	0.155
**Gender**						
Female-Male (Ref)	1.467	0.555–3.874	0.439	2.598	0.885–7.626	0.082
**Education**						
University/Postgraduate-Primary/middle school/high school (Ref)	0.896	0.347–2.308	0.820	2.41	0.741–7.839	0.144
**Employment Status**						
No-Yes (Ref)	1.028	0.403–2.624	0.953	1.047	0.364–3.013	0.931
**Marital Status**						
Married-Single (Ref)	2.349	0.378–14.580	0.359	3.633	0.549–24.016	0.181
**Do you have children?**						
Yes-No (Ref)	0.183	0.022–1.484	0.112	0.591	0.074–4.719	0.620
**Eating Behavior**						
Uncontrolled eating	0.990	0.967–1.013	0.397	0.994	0.969–1.020	0.691
Cognitive restraint	0.997	0.975–1.018	0.786	0.993	0.970–1.017	0.593
Emotional eating	0.995	0.982–1.008	0.517	1.004	0.990–1.018	0.561
Pittsburgh sleep quality (PSQ) score	1.342	1.073–1.678	0.010 *	1.485	1.157–1.905	0.002 *
Body Mass Index (BMI)	0.987	0.900–1.082	0.791	0.952	0.852–1.064	0.391
HEI-2015 score	1.042	1.000–1.086	0.047 *	1.030	0.985–1.077	0.193

Multinomial logistic regression, * *p* < 0.05 OR: Odds ratio. Dependent variable: Posttraumatic stress disorder. Independent variables: Age, gender, education, employment status, marital status, having children, eating behavior, sleep quality, body mass index, and diet quality. The independent variables—uncontrolled eating, cognitive restraint, emotional eating, sleep quality, body mass index, and diet quality—explain 12.1% of the variance in post-traumatic stress disorder (R^2^: 0.121).

## Data Availability

The raw data supporting the conclusions of this article will be made available by the authors on request.
